# Influence of Hashimoto thyroiditis on diagnosis and treatment of thyroid nodules

**DOI:** 10.3389/fendo.2022.1067390

**Published:** 2022-12-23

**Authors:** Linfeng Mao, Chunmei Zheng, Shengzhao Ou, Youwu He, Chuanjie Liao, Ganlu Deng

**Affiliations:** ^1^ Department of Hepatobiliary Surgery, The First Affiliated Hospital of GuangXi Medical University, Nanning, Guangxi, China; ^2^ Key Laboratory of Early Prevention and Treatment for Regional High Frequency Tumor (Guangxi Medical University), Ministry of Education, Nanning, Guangxi, China; ^3^ Guangxi Key Laboratory of Enhanced Recovery after Surgery for Gastrointestinal Cancer (Guangxi Medical University), Ministry of Education, Nanning, Guangxi, China; ^4^ Department of Pharmacy, The First Affiliated Hospital of Guangxi Medical University, Nanning, Guangxi, China; ^5^ Department of General Surgery, The Hepu People’s Hospital, Beihai, Guangxi, China; ^6^ Department of Oncology, The First Affiliated Hospital of Guangxi Medical University, Nanning, Guangxi, China

**Keywords:** thyroid cancer, Hashimoto thyroiditis, thyroid nodules, PTC, PTMC

## Abstract

**Background:**

As the prevalence of Hashimoto’s thyroiditis (HT) and thyroid cancer (TC) has been increasing dramatically in recent years, the association between the two diseases has been widely debated and studied. However, no consistent findings are available and the relationship remains controversial. In this study, we analyzed the influence of HT on the diagnosis and treatment of thyroid nodules and investigated the relationship between HT and TC.

**Methods:**

From Jan 2017 to Apr 2021, 4678 patients underwent thyroid surgery were collected. Of these patients, 440 were diagnosed with HT (242 nodular goiter (NG) with HT, 198 TC with HT). These patients were grouped when appropriate and the data from these patients were statistically analyzed by using SPSS and GraphPad Prism 6.

**Results:**

HT occurred in 198 of 1089 (18.2%) TC patients and 242 of 3589 (6.74%) patients without TC (*p*=0.000). In terms of the ultrasonography features, in the NG with HT group, 33.1% (80/242) of patients had fine calcification and 45.9% (111/242) of patients whose TI-RADS classification were > Level 3. In the isolated PTC group, 32.3% (2343/7260) LN were metastasis-positive while in the NG with HT group, only 26.0% (504/1939) LN were metastasis-positive (*P*=0.000). The proportion of PTMC was significantly higher (*P*=0.000), while the proportion of multifocal carcinoma was significantly lower when coexisting with HT (*P*=0.029). When comparing the data from the two groups diagnosed as PTMC coexisting with HT or not, there was no significant difference in the composition ratio of tumor number, LN metastasis, LN dissection area, regional LN metastasis and number of negative/positive LN (*P*=0.614, *P*=0.051, *P*=0.139, *P*=0.350, *P*=1.000 and *P*=0.333 respectively). In the MPTC group, 42.2% (872/2065) LN were metastasis-positive while in the MPTC with HT group, only 23.6% (50/212) LN were metastasis-positive (*P*=0.000).

**Conclusions:**

Our data suggested that HT is associated with an increased risk of developing TC but may be a protective factor against PTC progression and metastasis. The coexistence of HT affects the judgment of thyroid nodules by ultrasonography.

## Introduction

Hashimoto’s thyroiditis (HT), an autoimmune disease, is the most common cause of hypothyroidism and characterized by diffuse lymphocytic infiltration and gradual autoimmune which led to chronic inflammation and thyroid failure (1, 2). Increased serum anti-thyroid peroxidase antibodies (ATPO) and anti-thyroglobulin antibodies (ATG) are the main diagnostic indicators of Hashimoto’s disease. Under thyroid ultrasound, the main manifestation of HT is diffuse thyroid lesions, which can be further diagnosed by pathological examination (2). With the popularization of thyroid ultrasound examination and ultrasound-guided thyroid fine needle aspiration biopsy (FNAB), the detection rate of thyroid nodules continues increasing, as well as the increasing detection rate of thyroid cancer and HT (3, 4). The incidence of HT is reported to be 0.3-1.5 cases per 1000 individuals and is more prevalent in women (5, 6). In recent years, more and more studies have focused on the impact of HT on human health and found that HT is associated with an increased risk of cardiovascular disease and stroke (7–10).

Thyroid cancer (TC) is the most common endocrine malignancy, ranking in 9^th^ place in both sexes and 5^th^ in female for incidence (11, 12). The association between HT and TC has attracted increasing attentions due to the increasing incidence of TC together with HT. It has been hypothesized that HT is associated with an increased risk of papillary thyroid carcinoma (PTC). Despite the numerous studies assessing the association between HT and TC, no consistent findings are available and the relationship remains controversial (13–16). Due to the ongoing debate, we put forward this study aiming to determine the frequency of HT in patients diagnosed as benign as well as malignant thyroid nodules. Moreover, the patients who have nodular goiter (NG) with HT or TC with HT will be compared to those who have isolated PTC, in terms of patient characteristics, ultrasonography features, tumor type, tumor stage, tumor size and lymph node involvement. This study will help us understand the influence of HT on diagnosis and treatment of thyroid nodules and whether HT is associated with the increased risk and the higher stage of PTC.

## Materials and methods

From Jan 2017 to Apr 2021, 4678 patients underwent thyroid surgery at The First Affiliated Hospital of GuangXi Medical University and its interconnected hospital The Hepu People’s Hospital were enrolled in this study. Based on voluntary principles, the subjects were informed the purpose of this study and that they could freely decide whether they want to participate in the investigation or not. The subjects’ personal privacy and rights are fully protected. Research contents and results have no conflict of interest. Of these patients, 440 were diagnosed with HT (242 NG with HT, 198 TC with HT), all diagnoses are based on the final pathological diagnosis after surgery. For all patients, data were collected by retrospective chart review through electronic clinical records for patient general characteristics, clinicopathological characteristics, thyroid function, ATPO, ATG, ultrasonography features, nodule size, tumor stage, tumor number, presence of a goiter or malignancy, lymph nodes (LN) metastasis. The normal reference range of FT3、FT4、TSH was 2.8 to 7.1 pmol/L、12 to 22 pmol/L、0.27 to 4.20 μIU/ml respectively, and the normal reference ranges of ATPO and ATG were 0 to 34 IU/ml and 0 to 115 IU/ml respectively. If the patient’s test value is not within the normal range, make a corresponding clinical diagnosis, such as hyperthyroidism, hypothyroidism and HT. When the clinical diagnosis is inconsistent with the pathological diagnosis, the pathological diagnosis shall prevail. The staging of TC is based on the eighth edition AJCC TNM classification for thyroid carcinoma (AJCC 8th ed., 2017).

### Statistical analysis

Statistical analyses were performed with SPSS software program (version 21.0; IBM Corporation) and GraphPad Prism 6 (San Diego, CA). In analysis of these data, t-test, Chi-square test, Fisher’s exact test, and one-way analysis of variance (ANOVA) was utilized when appropriate. With regard to the results, *P*<0.05 was considered to be statistically significant. The collection of patients’ data and subsequent analysis was approved by the Research Ethics Committee of The First Affiliated Hospital of GuangXi Medical University and The Hepu People’s Hospital.

## Results

### Patient characteristics of nodular goiter with HT and thyroid cancer with HT

Overall, of the 4678 patients, 9.4% (440/4678) were diagnosed with HT (242 NG with HT, 198 TC with HT) and 23.3% (1089/4678) were diagnosed with TC based on final pathology. Of the 1089 patients who were diagnosed with TC, 18.2% (198/1089) were together with HT, while, of the 3589 patients who were diagnosed with benign thyroid nodule, only 6.74% (242/3589) were together with HT, which indicates that the proportion of TC combined with HT is higher and HT may be a risk factor for TC. Of the 440 patients who were diagnosed with HT, 90.5% (398/440) were female while only 9.5% (42/440) were male, which means that HT is more prevalent in women as well as TC. When comparing patients who were diagnosed as NG with HT to those were diagnosed as TC with HT (**Table 1**), the mean age of the TC with HT group was younger than the NG+HT group (37.80 ± 11.49 vs 47.14 ± 11.50, *P*=0.000), which may be due to HT can affect and improve the TI-RADS grading of thyroid nodules in the diagnosis process that lead to more active and earlier treatment measures for patients with HT and thyroid nodules, and the proportion of males is higher in the TC+HT group (16/242 vs 26/198, *P*=0.021). Although the nodule sizes of the NG with HT group appeared to be slightly smaller than those of TC with HT group (1.29 ± 0.97cm vs. 1.41 + 1.05cm), this difference was not significant (*P*=0.2142). In terms of thyroid function, there was no significant difference in the composition ratio between the two groups (P=0.126). There was no significant difference in the preoperative mean ATG value (347.21 ± 436.95 vs 357.95 ± 370.34) between NG with HT group and TC with HT group (P=0.7839). Similarly, there was no significant difference in the preoperative mean ATPO value (150.78 ± 160.09 vs 161.15 ± 162.63) between the two groups (P=0.5025). In addition, in terms of the composition ratio of ATG/ATPO positive rate, there was no significant difference between the two groups (*P*=0.674). Of the 440 patients who were diagnosed with HT based on final pathology, 13.6% (60/440) of patients whose serum ATG and ATPO were double negative, which suggests that ATG and ATPO negativity cannot rule out the diagnosis of HT.

**Table 1:** Patient characteristics of nodular goiter with HT and TC with HT.

		NG+HT(n=242)	TC+HT (n=198)	t/χ2	*P*-value
Age (years, mean±SD)		47.14±11.50	37.80±11.49	8.479	0.000^*^
Gender	Male	6.6% (16/242)	13.1% (26/198)	5.361	0.021^*^
	Female	93.4% (226/242)	86.9% (172/198)		
Tumor size (cm, mean±SD)		1.29±0.97	1.41+1.05	1.244	0.2142
Thyroid function	Normal	64.9% (157/242)	73.2% (145/198)	4.141	0.126
Hyperthyroidism		11.6% (28/242)	10.6% (21/198)		
	Hypothyroidism	23.5% (57/242)	16.2% (32/198)		
ATG (IU/mL, mean±SD)		347.21±436.95	357.95±370.34	0.2745	0.7839
ATPO (IU/mL, mean±SD)		150.78±160.09	161.15±162.63	0.6712	0.5025
ATG、ATPO	Double positive	42.6% (103/242)	48.5% (96/198)	2.339	0.674
	Only ATG positive	21.0% (51/242)	18.7% (37/198)		
	Only ATPO positive	19.0% (46/242)	18.7% (37/198)		
	Double negative	15.3% (37/242)	11.6% (23/198)		
	Untested	2.1% (5/242)	2.5% (5/198)		

ATPO, anti-thyroid peroxidase antibodies; ATG, anti-thyroglobulin antibodies; *p<0.05.

### Ultrasonography features of nodular goiter with HT and TC with HT

When comparing the ultrasonography features of patients who were diagnosed as NG with HT to those were diagnosed as TC with HT, in terms of diffuse thyroid gland and tumor number, there was no significant difference between the two groups (**Table 2**, *P*=0.241 and *P*=0.383 respectively). There were significant differences between the two groups (*P*=0.000) in the appearance of calcifications, in the TC with HT group, the main manifestations of ultrasonography were fine calcifications which accounted for 88.4% (175/198), and no calcifications only accounted for 4.5% (9/198), on the contrary, in the NG with HT group, no calcifications accounted for 55.4% (134/243), which indicates that fine calcifications are an important indicator for differentiating benign and malignant thyroid nodules. In terms of TI-RADS classification, in the NG with HT group, 45.9% (111/242) of patients were > Level 3, while in the TC with HT group, 91.4% (181/198) of patients were > Level 3, there were significant differences between the two groups (*P*=0.000). These suggested that HT may affect the TI-RADS classification of thyroid nodules, and the TI-RADS classification of thyroid nodules with HT may be higher, thereby influencing clinical decisions.

**Table 2:** Ultrasonography features of nodular goiter with HT and TC with HT.

		NG+HT (n=242)	TC+HT (n=198)	t/χ2	*P*-value
Diffuse thyroid gland	YES	30.6% (74/242)	35.9% (71/198)	1.374	0.241
	NO	69.4% (168/242)	64.1% (127/198)		
Number of tumors	Single	26.9% (65/242)	23.2% (46/198)	0.76	0.383
	Multiple	73.1% (177/242)	76.8% (152/198)		
Calcification	Fine calcification	33.0% (80/242)	88.4% (175/198)	142.38	0.000^*^
	Coarse calcification	11.6% (28/242)	7.1% (14/198)		
	No calcification	55.4% (134/242)	4.5% (9/198)		
TI-RADS classification	≤ Level 3	54.1% (131/242)	8.6% (17/198)	101.204	0.000^*^
	> Level 3	45.9% (111/242)	91.4% (181/198)		

*p<0.05.

### Patient characteristics of isolated TC and TC with HT

Of the 1089 patients who were diagnosed with TC, 18.2% (198/1089) were together with HT, 81.8% (891/1089) were diagnosed as isolated TC without HT. When comparing the patient characteristics of isolated TC to those were diagnosed as TC with HT, in terms of age, tumor size and tumor stage, there was no significant difference between the two groups (**Table 3**, *P*=0.0687, *P*=0.890 and *P*=0.473 respectively). In isolated TC group, 27% (241/891) were male and 73% (650/891) were female, while in TC with HT group only 13.1% (26/198) were male, there were significant differences between the two groups (**Table 3**, *P*=0.000) in terms of gender composition. In terms of tumor type, there were also significant differences between the two groups (**Table 3**, *P*=0.000), in TC with HT group, the proportion of FTC and MTC increased significantly, while, in isolated TC group, the proportion of ATC was higher than that in the TC with HT group, however, due to the limited sample size, there are too few cases diagnosed as MTC/ATC by final pathology, so the relationship between HT and MTC/ATC cannot be clarified and need to be further confirmed by expanding the sample size.

**Table 3:** Patient Characteristics of isolated TC and TC with HT.

		Isolated TC (n=891)	TC+HT (n=198)	t/χ2	*P*-value
Age (years, mean±SD)		39.60±12.80	37.80±11.49	1.822	0.0687
Gender	Male	27.0% (241/891)	13.1% (26/198)	16.954	0.000^*^
	Female	73.0% (650/891)	86.9% (172/198)		
Tumor size (cm, mean±SD)		1.40±0.89	1.41±1.05	0.1382	0.89
Tumor type	PTC	98.9% (881/891)	95.5% (189/198)	22.375	0.000^*^
	FTC	0.6% (5/891)	2% (4/198)		
	MTC	0.1% (1/891)	2.5% (5/198)		
	ATC	0.4% (4/891)	0% (0/198)		
Tumor stage	I-II stage	94.7% (844/891)	96.0% (190/198)	0.515	0.473
	III-IV stage	5.3% (47/891)	4.0% (8/198)		

*p<0.05.

### Patient characteristics of isolated PTC and PTC with HT

Of the 1089 patients who were diagnosed with TC, PTC was accounted for 98.3% (1070/1089), therefore, in order to explore the effect of HT on PTC progression and metastasis, we conducted a comparative analysis between the isolated PTC group and the PTC with HT group. As can be seen from **Table 4** that there was no significant difference between the two groups in the composition ratio of lymph node dissection, lymph node metastasis, lymph node dissection area and regional lymph node metastasis (**Table 4**, *P*=0.376, *P*=0.130, *P*=0.601, and *P*=0.850 respectively). In the isolated PTC group, a total of 7260 lymph nodes were dissected from 727 patients, of which 32.3% (2343/7260) were metastasis-positive and 67.7% (4917/7260) were metastasis-negative, while, in the PTC with HT group, a total of 1939 lymph nodes were dissected from 161 patients, of which 26% (504/1939) were metastasis-positive and 74% (1435/1939) were metastasis-negative, there were significant differences between the two groups (**Table 4**, *P*=0.000), which indicates that PTC patients with coexisting HT were likely less frequent nodal metastases. There was a significant difference between the two groups in terms of the composition ratio of PTMC and multifocal carcinoma between the two groups (**Table 4**, *P*=0.000 and *P*=0.029 respectively), compared with the PTC with HT group, in the isolated PTC group, the proportion of PTMC was significantly lower, while the proportion of multifocal carcinoma was significantly higher, which indicated that PTC patients with coexisting HT were likely less aggressive disease.

**Table 4:** Patient Characteristics of isolated PTC and PTC with HT.

		Isolated PTC (n=881)	PTC+HT(n=189)	t/χ2	*P*-value
LN dissection	YES	82.5% (727/881)	85.2% (161/189)	0.783	0.376
	NO	17.5% (154/881)	14.8% (28/189)		
LN metastasis	YES	65.3% (475/727)	59.0% (95/161)	2.298	0.13
	NO	34.7% (252/727)	41.0% (66/161)		
LN dissection area	Central district	63.7% (463/727)	61.5% (99/161)	0.274	0.601
	Central + side area	36.3% (264/727)	38.5 (62/161)		
Regional LN metastasis	Central district	59.2% (281/475)	58.9% (56/95)	0.325	0.85
	Central + side area	36.8% (175/475)	35.8% (34/95)		
	Only side area	4.0% (19/475)	5.3% (5/95)		
Number of N/P LN	Total	7260	1939		
	Negative	67.7% (4917/7260)	74.0% (1435/1939)	28.24	0.000^*^
	Positive	32.3% (2343/7260)	26.0% (504/1939)		
PTMC	YES	6.9% (61/881)	20.6% (39/189)	34.529	0.000^*^
	NO	93.1% (820/881)	79.4% (150/189)		
Multifocal carcinoma	YES	18.8% (166/881)	12.2% (23 /189)	4.764	0.029^*^
	NO	81.2% (715/881)	87.8% (166/189)		

*p<0.05.

### Patient characteristics of PTMC and PTMC with HT

PTMC refers to PTC with a tumor diameter no more than 1 cm, also known as occult PTC, which belongs to the very early stage of cancer. From the data presented above, the proportion of PTMC was significantly difference between the isolated PTC group and the PTC with HT group. Therefore, we further grouped and compared patients’ characteristics who were diagnosed with PTMC and PTMC together with HT. As showed in **Table 5**, there is no significant difference between the two groups in age and the composition ratio of tumor number, LN metastasis, LN dissection area, regional LN metastasis and number of negative/positive LN (**Table 5**, *P*=0.614, *P*=0.051, *P*=0.139, *P*=0.350, *P*=1.000 and *P*=0.333 respectively). In patients of PTMC group, 19.7% (12/61) were male and 80.3% (49/61) were female, while in PTMC with HT group only 5.1% (2/39) were male, there were significant differences between the two groups (**Table 6**, *P*=0.039) in terms of gender composition. These indicated that co- existing with HT at the early stage of the tumor does not affect the lymph nodes metastasis ability of the tumor, however, due to the limitation of sample size which were diagnosed as PTMC or PTMC with HT, this conclusion needs to be confirmed by further expanding the sample size.

**Table 5:** Patient Characteristics of PTMC and PTMC with HT.

		PTMC(n=61)	PTMC+HT(n=39)	t/χ2	*P*-value
Age (years, mean±SD)		39.25±10.84	38.05±12.63	0.506	0.614
Gender	Male	19.7% (12/61)	5.1% (2/39)	4.18	0.041^*^
	Female	80.3% (49/61)	94.9% (37/39)		
Tumor number	Single	73.8% (45/61)	89.7% (35/39)	3.794	0.051
	Multiple	26.2% (16/61)	10.3% (4/39)		
LN metastasis					
	Not cleaned	26.2% (16/61)	28.2% (35/39)	3.945	0.139
	NO	44.3% (27/61)	59.0% (23/39)		
	YES	29.5% (18/61)	12.8% (5/39)		
LN dissection area	Total	45	28		
	Central district	82.2% (37/45)	92.9% (26/28)	0.874	0.35
	Central + side area	17.8% (8/45)	7.1% (2/28)		
Regional LN metastasis	Total	18	5		
	Central district	83.3% (15/18)	80.0% (4/5)	0	1
	Central + side area	16.7% (3/18)	20.0% (1/5)		
Number of N/P LN					
	Total	242	129		
	Negative	84.7% (205/242)	88.4% (114/129)	0.936	0.333
	Positive	15.3% (37/242)	11.6% (15/129)		

LN, lymph nodes; PTMC, papillary thyroid microcarcinoma; N/P, Negative/Positive; *p<0.05..

### Patient characteristics of MPTC and MPTC with HT

From the data presented above, we found that the proportion of multifocal PTC (MPTC) was significantly difference between the isolated PTC group and the PTC with HT group. Therefore, we further grouped and compared patients’ characteristics who were diagnosed with MPTC and MPTC together with HT. In terms of age, the composition ratio of LN dissection area and regional LN metastasis, there was no significant difference between the two groups (**Table 6**, *P*=0.0945, *P*=0.122 and *P*=0.732 respectively). In MPTC group, 19.9% (33/166) were male and 80.1% (133/166) were female, while in MPTC with HT group 100% (23/23) were female, there were significant differences between the two groups (**Table 6**, *P*=0.039) in terms of gender composition, which mean that MPTC with HT is more prevalent in women. In terms of LN metastasis, in the MPTC group, of 166 patients, 156 patients underwent lymph node dissection and 69.9% (109/156) had lymph node metastases, while, in the MPTC with HT group, of 23 patients, 17 patients underwent lymph node dissection and 52.9% (9/17) had lymph node metastases, there was no significant difference between the two groups (*P*=0.155). However, in the MPTC group, a total of 2065 lymph nodes were dissected from 156 patients, of which 42.2% (872/2065) were metastasis-positive and 57.8% (1193/2065) were metastasis-negative, while, in the MPTC with HT group, a total of 212 lymph nodes were dissected from 17 patients, of which 23.6% (50/212) were metastasis-positive and 76.4% (162/212) were metastasis-negative, there were significant differences between the two groups (**Table 6**, *P*=0.000), which indicates that MPTC patients with coexisting HT were likely less frequent nodal metastases, however, due to the limitation of sample size which were diagnosed as MPTC with HT, this conclusion needs to be confirmed by further expanding the sample size.

**Table 6:** Patient Characteristics of MPTC and MPTC with HT.

		MPTC(n=166)	MPTC+HT(n=23)	t/χ2	*P*-value
Age (years, mean±SD)		40.05±11.89	35.65±10.80	1.681	0.0945
Gender	Male	19.9% (33/166)	0.0% (0/23)	4.246	0.039^*^
	Female	80.1% (133/166)	100% (23/23)		
LN metastasis					
	Not cleaned	6.0% (10/166)	26.1% (6/23)	12.173	0.002^*^
	NO	28.3% (47/166)	34.8% (8/23)		
	YES	65.7% (109/166)	39.1% (9/23)		
LN dissection area					
	Central district	57.1% (89/156)	76.5% (13/17)	2.389	0.122
	Central + side area	42.9% (67/156)	23.5% (4/17)		
Regional LN metastasis					
	Central district	45.9% (50/109)	55.6% (5/9)	0.313	0.732
	Central + side area	54.1% (59/109)	44.4% (4/9)		
Number of N/P LN					
	Total	2065	212		
	Negative	57.8% (1193/2065)	76.4% (162/212)	27.731	0.000^*^
	Positive	42.2% (872/2065)	23.6% (50/212)		

MPTC, multifocal PTC; *p<0.05..

## Discussion

The exact etiology of HT has not been fully elucidated, genetic susceptibility, immune disorders, environmental factors and epigenetic factors affect the occurrence and development of it. Evidence implicates that avoiding high iodine intake, appropriate selenium, vitamin D and iron supplementation have certain effects on the treatment of HT, and lead to a reduction of serum ATG/ATPO level (17, 18). Since the initial description of the relationship between HT and PTC by Dailey (19), the association between the two diseases has been widely debated and studied. Because of HT is an autoimmune disease which led to chronic inflammation of thyroid, it has been hypothesized that HT is associated with an increased risk of TC according to the “inflammation-tumorigenesis” theory (20), and numerous studies had investigated this hypothesis. However nearly all studies have been confounded by imprecise metrics, substantial selection bias and retrospective analysis (1, 21–23). Conflicting reports continue to emerge, Holm et al. found that HT patients had no obvious risk of thyroid cancer (24). Xu et al. found that HT is a “double-edged sword” in TC patients (15), therefore the relationship between the two diseases remains controversial. In our study, we found that of the 1089 patients who were diagnosed with TC, 18.2% were together with HT, while, of the 3589 patients who were diagnosed with benign thyroid nodule, only 6.74% were together with HT, which indicates that the proportion of TC combined with HT is higher and suggests an obvious relationship between HT and TC, HT may be an important risk of TC.

Definition of HT requires histopathological diagnosis, however, currently other means of preoperative non-invasive diagnosis had been proven highly predictive of the disease, including the detection of serum ATG and ATPO, as well as the identification of a diffusely heterogeneous parenchyma upon sonographic imaging of the thyroid gland (1). However, in our study, of the 440 patients who were diagnosed with HT based on final pathology, 13.6% of patients whose serum ATG and ATPO were double negative, which suggests that ATG and ATPO negativity cannot rule out the diagnosis of HT. What’s more, when we analyzed the ultrasonography features of nodular goiter with HT and thyroid cancer with HT, we found that only 33% of patients had explicitly reported diffusely heterogeneous parenchyma of the thyroid gland. Therefore, the diagnostic methods used alone have certain false negatives, and their combined application can improve the diagnostic accuracy of HT. Furthermore, Nathalie et al. found that the diagnostic evaluation of thyroid nodules is affected by the presence of HT, even when the nodule is nonmalignant, and a significantly higher risk of indeterminate cytology should be expected (1). TI-RADS is founded on the evaluation of US features in five categories—composition, echogenicity, shape, margin, and echogenic foci, which is an important method for preoperative assessment of benign and malignant thyroid nodules, and has important reference significance for artificial intelligence (AI) to realize the characterizations of thyroid nodules and for the treatment of thyroid nodules (25, 26). It has been reported that the proportion of TI-ARDS>3 in benign thyroid nodules was found to be about 9% assessed by thyroid ultrasound (27). However, we found that the TI-ARDS classification of 45.9% patients who diagnosed NG with HT were > Level 3, which suggested that HT may affect the TI-RADS classification of thyroid nodules, and the TI-RADS classification of thyroid nodules with HT may be higher, thereby influencing clinical decisions.

Tumor diameter, lymph node metastasis and multifocal carcinoma are all the main risk factors for PTC progression and recurrence. In our study, the proportion of PTMC was significantly higher when coexisting with HT. It may be because HT patients are prone to anxiety and frequently undergo thyroid ultrasonography, which is more likely to detect malignant thyroid nodules early. It remains unclear whether HT is a risk factor for lymph node metastasis of TC. Studies have found that the lymphocytic infiltration of HT may be an immunologic response with a cancer-impeding effect and PTC patients with coexisting HT are likely to be with less aggressive disease, less frequent nodal metastases, less likely to develop recurrence, and have a higher survival rate (28–32). However, some studies have also found that HT is not associated with lymph node metastasis of TC and promotes the progression of TC (33–35). Our data showed that PTC patients with HT to have a lower prevalence of lymph-node metastasis and HT may be a protective factor against PTC metastasis. In terms of multifocal carcinoma, our study showed that the proportion of multifocal carcinoma was significantly lower when coexisting with HT, which conclusion is consistent with the conclusions reached by Cappellacci et al. (36) but not by Xu et al. (15).

Our study explored the influence of Hashimoto thyroiditis on diagnosis and treatment of thyroid nodules and mainly analyze the relationship between TC and HT, but had two main limitations. First, the study was retrospective: more real-world studies and prospective studies are needed to obtain more accurate conclusions. Second, the sample size of this study is small, more samples need to be collected to reduce experimental errors and draw more accurate conclusions.

## Conclusions

HT increases the risk of TC but is a protective factor against PTC progression and metastasis.

## Data availability statement

The raw data supporting the conclusions of this article will be made available by the authors, without undue reservation.

## Author contributions

LM and CZ conceived this study, GD directed the study. LM and CZ performed most of the experiments. LM drafted the manuscript. LM, CZ and SO collected and analyzed the data and performed the statistical analyses. YH and CL participated in some experiments. GD and LM provided critical intellectual revision. GD provided financial support. All authors contributed to the article and approved the submitted version.
